# Challenges in setting up a primary human papillomavirus-DNA testing facility in a lower and middle income country: lessons learned from a pilot programme

**DOI:** 10.3332/ecancer.2022.1492

**Published:** 2022-12-19

**Authors:** Kavitha Dhanasekaran, Harki Tamang, Sangeeta Pradhan, Rinzing Lhamu, Roopa Hariprasad

**Affiliations:** 1 Division of Clinical Oncology, ICMR-National Institute of Cancer Prevention and Research, Noida, Uttar Pradesh 201301, India; 2 Department of Health and Welfare, Government of Sikkim, Gangtok, Sikkim 737101, India; a https://orcid.org/0000-0002-3756-3419; b https://orcid.org/0000-0003-2032-3432

**Keywords:** primary HPV screening, cervical cancer, HPV DNA testing facility, challenges, LMIC

## Abstract

**Introduction:**

Detection of high-risk human papillomavirus (hrHPV) is the most sensitive test for the screening of cervical cancer. Although most high-income countries have adopted this strategy in their screening programme, there are a lot of barriers in low and middle-income countries (LMICs) in setting up this facility for screening. The lessons learned based on this experience can be useful for other LMICs in their first steps to integrate HPV testing into a screening programme.

**Methods:**

HPV testing using self-sampling was offered to eligible women residing in one district of Sikkim state. To implement the same, a testing laboratory was set up in the district and the challenges faced are listed.

**Results:**

The cost of testing equipment, sampler and cold storage was beyond the budget capping. Setting up of the HPV testing lab accessible to study sites and referral centre was a difficult decision to make. Training the health care providers in their proficiency in triaging and treatment was challenging. Coordinating with community health workers and beneficiaries for effective screening and establishing referral linkages was not easy, as we expected. The cost of transportation, consumables and contingencies was higher due to the difficult terrain.

**Conclusion:**

The cost of the equipment and consumables for primary HPV screening can be reduced in bulk purchases through negotiations. Adequate knowledge of the terrain and economic implications of the area of interest is crucial during the budgeting of the programme. Collaborating with the state government, integration with the existing health system and repurposing the available resources are key for success. The barriers faced during implementation are stepping stones for improvement.

## Introduction

Cervical cancer is the second most common cancer in Indian women, resulting in more than 1,23,000 new cases in 2020 [1]. Of the incident cases in India, approximately 60% die due to cervical cancer, accounting for nearly one-fourth of the global mortality due to cervical cancer [1]. There is a paradigm shift in India from opportunistic to population-based cancer screening with the Ministry of Health and Family Welfare of the Government of India launching the operational framework in 2017, which consisted of screening and early detection of three preventable cancers of oral, breast and cervical cancer [2]. Although visual inspection with acetic acid (VIA), screening method adopted for cervical cancer is evidence based, there are lots of criticisms raised on the feasibility of implementing this in the field by health care workers [3]. Moreover, lack of clear framework defining key milestones and deliverables and performance evaluation indicators might be a hindrance in measuring the impact of cancer control plan. To align with the recent call to action by World Health Organization (WHO) to eliminate cervical cancer, India should introduce screening of women with a highly sensitive test. Human papillomavirus (HPV)-DNA testing is recommended as the most effective strategy for cervical cancer screening [4]. Despite the high incidence of cervical cancer in low & middle-income countries (LMICs), there are several impediments for implementing primary HPV screening in a real-life setting with limited resources [5]. The major hurdle is setting up an HPV testing facility due to the higher up-front costs for the equipment and supplies [6]. Since the study is ongoing, in this article, we have shared our experience with setting up of HPV testing facility which was the primary step and the most difficult part of our implementation research study in one of the districts of Sikkim, India and the results of the study will be disseminated on its completion. The lessons-learned during the implementation process are enumerated to assist other states and LMICs to replicate this model and contextualise it to their respective settings.

## Methods

Our implementation research study was conducted with the objective of assessing the feasibility of introducing primary HPV DNA testing on self-collected samples in one district of Sikkim with the state government as a part of population-based screening programme. This north-eastern Indian state’s terrain is mainly mountainous and is devoid of any plain lands making it very difficult to commute from one place to other. The capital of the state, Gangtok is situated in East district. Sikkim is the first ever state in India to include HPV vaccination for eligible girls in their immunisation schedule since 2018 [7]. Considering the ease of coordination, East district was chosen for implementing primary HPV-DNA screening using self-sampling and setting up of testing facility.

**Figure 1.**
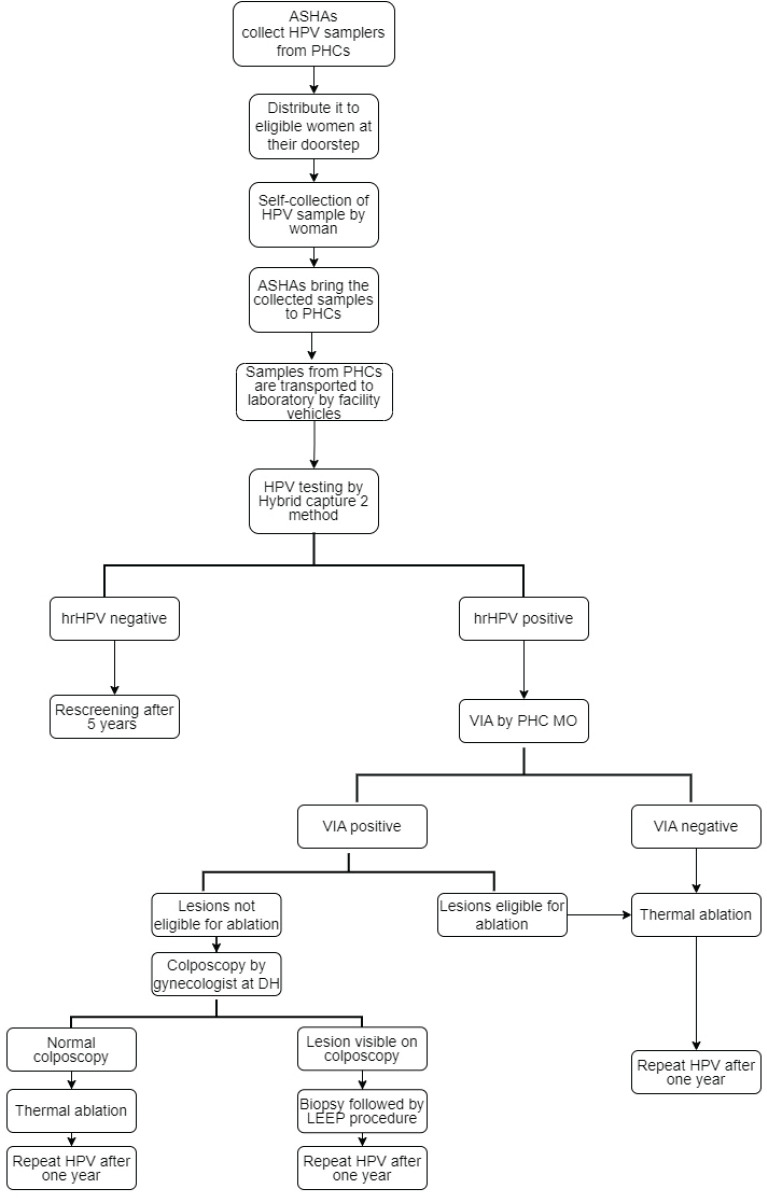
Flow-chart illustrating the study design. ASHA, Accredited Social Health Activist; DH, District hospital; hrHPV, High-risk HPV; MO, Medical officer; PHC, Primary health centre; LEEP, Loop electrosurgical excision procedure; VIA, Visual inspection using acetic acid.

The detailed flow chart on the study design is provided in Figure 1. Accredited Social Health Activists (ASHAs) collected HPV samplers from primary health centres (PHCs) and took them along with them during their home visits in the community. ASHAs explained the steps of vaginal sample collection to eligible women with the help of Information, Education and Communication (IEC) materials in the form of charts and videos developed in this programme. Women collected the sample in the privacy of another room or toilet and handed over the collected specimen to ASHA worker either on the same day or within a week of collection. The home collected samples were deposited to the respective PHCs by ASHA workers and the samples were in turn transported to the testing lab on a weekly basis through the facility vehicle. On rare occasions, in case of non-availability of the facility vehicle, the samples were transported through hired vehicles to avoid delay in the sample transportation.

The test results were communicated to the individuals through the ASHA workers (hard copy) and soft copy sent to the participants on their mobile number through WhatsApp. The reporting time ranged from 5 to 12 days of sample collection. In addition to this, all women with HPV positive report were telephonically contacted by lab technician and requested to visit their respective PHCs for further evaluation.

## Results

A testing facility was set-up at the district hospital, Singtam, East district Sikkim to test the collected samples for high-risk HPV_DNA. The step-wise process in setting up of the facility along with the challenges faced during the implementation of the same are elaborated below.

### Procurement of hybrid-capture 2 (HC2) testing device, reagents and sample collection kits

The HC2 testing device and 6,000 collection kits procurement was the first step in setting up the facility at the study site. The high cost of the equipment seemed to be a deterrent for implementing HPV based screening in population-based setting. After multiple rounds of negotiations, the manufactures agreed to reduce the cost to 50% of the actual proposed price by donating 5,000 additional testing kits. This opened a great opportunity to screen more eligible women.

The device comes with few accessories such as water bath, vortex, etc., that are not included when the final price is quoted. Hence, these were not budgeted under the programme funds. The state government negotiated with the suppliers to provide it as complimentary along with the device.

Due to COVID-19 pandemic, all the process including the receipt of the consumables and equipment were delayed which led to delay in start of sample collection and testing. Due to the budget capping, the funds allotted for equipment and the testing kits were not sufficient to purchase deep freezer (−20°C) to store the tested HPV samples. However, the district hospital (DH) blood bank agreed to share their deep freezer for this purpose.

### Setting up of HPV-DNA testing facility

The lab technician recruited for this study has ‘Bachelor degree in Medical laboratory technology’ with 3 years of work experience. Soon after the recruitment of technician, a 3-day training was provided by expert master trainer from the manufacturer of Hybrid Capture device. Since there was a gap of 3 months from the recruitment to the initiation of the study, a refresher training of 2 days duration was provided by the same master trainer. Before testing the actual samples, multiple dummy tests were run by the lab technician under the supervision of the master trainer. As per the referral linkage in the health system, the DH located at Singtam, caters the PHCs from east district. Our implementation sites being urban PHC – Gangtok, and Sang PHC from east, we identified a lab space and set-up the HPV testing at the DH. This served as the referral centre for diagnostic and treatment centre for the study participants. During our second dry run of the samples, there was power failure for more than 8 hours and we learnt that due to old and damaged power lines major maintenance work in progress in the hospital which was expected to last for few months. To have power backup was not possible because of the maintenance works and budget constraints.

The distance from the implementation sites to DH Singtam ranged from 13 to 27 kms which translates to approximately 1 to 2 hours of travel in hilly roads in suitable weather conditions. Commuting samples on a daily basis was cumbersome and the transportation charges were exorbitantly high due to difficult terrain and non-reliable public transport. Hence, shifting the HPV DNA testing clinic closer to collection sites seemed a feasible option and therefore the lab was set up *de novo* at Urban Primary Health Centre (UPHC) Gangtok which is accessible for the study sites.

Quality control: Retesting of 10% random positive and negative samples was done by the expert master trainer every 3 months as a part of quality control process.

### Strengthening the referral centres confirmation of diagnosis and treatment

The referral centre for diagnostic procedure with colposcopy examination and the treatment using excisional method, Loop Electrosurgical Excisional Procedure (LEEP) unit is at the DH, Singtam. Since the colposcopy examination procedures were started for the first time at DH, the colposcopy and treatment clinic were set-up afresh. The State Government of Sikkim (SGS) procured the colposcope and LEEP device from the state health budget and handheld thermo-coagulator in collaboration with a non-governmental organisation. The gynaecologists and the support staff at the district hospital were trained in colposcopy examination and LEEP procedure through hybrid training module (online followed by hands-on training).

However, while performing procedures, there were multiple queries from the healthcare providers in interpretation of the results and decision-making regarding treatment. Hence, refresher training was provided to the team along with hand-holding while they performed the colposcopy and LEEP procedures. Despite our efforts to setup the referral linkage to DH, Singtam, the participants preferred visiting the super speciality hospital (SSH) located at Gangtok considering the accessibility and distance. Hence, we trained the gynaecologists and the support staff in colposcopy, thermal ablation and LEEP procedures and the SGS strengthened the facility in terms of consumables and equipment. However, SSH being the only public tertiary care centre in the entire state, it is overburdened with referrals for emergencies and surgeries. Healthcare providers were not able to cater the referrals for colposcopy and treatment of precancers. As a measure of reducing the lost to follow-up of the screen test positive individuals, providing access to treatment to the participants and to reduce the burden of unnecessary referrals to the DH, all HPV positives were invited to the respective PHCs for triaging using VIA and treatment with thermal ablation. The participants who required excisional treatment with LEEP were referred to DH and the SSH.

Hence, medical officers and the support staff were trained at the implementation sites in VIA test and thermal ablation procedure to triage and treat. After few months of initiation of the study, the trained medical officers from both the implementation sites were transferred to different facilities and new doctors replaced them. Keeping attrition of doctors in mind, training courses were made more frequent for newly joined medical officers replacing the already trained medical officers. Considering the sustainability of the programme after the completion of the study, we trained healthcare providers from other facilities which include 12 gynaecologists, 30 medical officers, 40 staff nurses/mid-level healthcare providers and 74 ASHA workers.

### Coordinating with ASHA workers for sample collection

Community health workers in India are called Accredited Social Health Activists (ASHAs) who form an important interface between the community and the public health system. The role of ASHAs in this programme is to counsel eligible women from the community to undergo cervical cancer screening, deliver the testing kit to them, collect the kits after sample is collected by women and bring them during their monthly review meetings to UPHC Gangtok. However, the number of samples that ASHAs managed to bring was not as desired. With other responsibilities and priorities, ASHAs found it difficult to spend more time in counselling women. Moreover, ASHAs are a non-salaried voluntary cadre of health staffs whose main stay of income is from their performance-based incentives. This makes them focus more towards maternal and child health programmes which has well-defined incentives for each activity.

Some of the alternate methods were tried to increase the screening coverage such as integrating the community awareness and sample collection with other programmes which included village health, sanitation and nutrition committee meetings.

## Discussion

The lessons learned during the implementation of pilot programme have been summarised in Table 1. We attempted to understand the experiences from other LMICs on implementing primary HPV-DNA testing in population-based cancer screening. We found studies that tested the feasibility of using HPV-DNA testing for cervical cancer screening in LMICs as standalone projects [8–10]. However, none of these studies discussed about the real-time challenges in setting-up of a lab and implementing HPV-DNA testing at community level. Majority of the studies report the beneficiary level barriers which included wrong perceptions of women regarding self-collection, lack of cervical cancer screening knowledge, constraints in providing quality sample, follow-up of screen positive women and socio-economic factors [11–13]. Some of the solutions suggested to overcome fear, shyness and lack of knowledge leading to misconceptions were to adopt community-engaged approach to increase community participation, to allow women to touch and feel the sampling brush to convince her that it is soft and would not hurt, more educational pictures and use of a doll to simulate the procedure, organise community awareness programmes to dispel fear and misconceptions, health education sessions and counselling women regarding the follow-up visits [14].

To overcome the administrative related challenges, it is imperative for the programme managers and state health officials to take the onus of the implementation. Cervical cancer screening programme falls under a larger umbrella of National Programme for Prevention and Control of Cancer, Diabetes, Cardiovascular diseases and Stroke (NPCDCS) programme for optimal utilisation of resources. Since all the components of NPCDCS programme share the same infrastructure and human resources, intersectoral coordination and multistakeholder involvement play an important role [15].

Our experiences are different due to the uniqueness of the study which is primary HPV-DNA testing through self-sampling that is being implemented for the first time in the country as a part of population-based cancer screening programme by a state.

## Conclusion

The cost of the equipment and consumables for primary HPV screening can be reduced in bulk purchases through negotiations. Adequate knowledge of the terrain and economic implications of the area of interest is crucial during the budgeting of the programme. Collaborating with the state government, integration with the existing health system and repurposing the available resources are key for success. Eventually when countries consider replacing VIA or cytology-based screening with primary HPV testing, these experiences will be beneficial during the execution of the programme. Challenges in the initial stages of implementation of any new programme are opportunities for a better beginning!

## Conflicts of interest

The authors declare that they have no conflicts of interest or personal relationships that could have appeared to influence the work reported in this paper.

## Trial registration

Trial registered in Clinical Trial Registry of India (CTRI). Trial No CTRI/2020/11/029419.

## Funding

This work was supported by the Indian Council of Medical Research (Award No No.5/13/3/RHP/ICRC/2020/NCD-III)

## Ethics approval from both the sites

1. ICMR-NICPR (ref No NICPR/IEC/2019/008) dated 18 December 2019
2. Sikkim state govt (ref No 02/IEC/NSTNMH/20) dated 25 February 2020

## Figures and Tables

**Table 1.** Lessons learned during the implementation of pilot programme.

Process	Lessons learned
Procurement of equipment and supplies	Negotiation with vendor for the cost of the equipment and the consumables when its bulk purchase for population-based implementation of screening will slash the unit cost of each test
Identifying a place to set-up the testing facility	Necessity of identification of right place for setting-up of the equipment in terms of uninterrupted power supply and proximity to the sample collection
Capacity building	Initial hand-holding of health care providers in performing screening, triaging and treatment till they acquire necessary skills and confidence is imperative Retraining of human resources for upgrading the required knowledge and skills is vital
Integration with the existing services	The key for the sustainability of the programme is the integration with other govt led programmes and engaging the existing health system and healthcare providers for the implementation of the screening, diagnostics and treatment. Collaboration of State government with non-governmental organisations in implementation of population-based screening programme
Collection of samples	Challenges in motivation of community health workers and the beneficiaries compel for alternative approaches to engage the community in HPV-self sampling There is a need for incessant behaviour change communication about screening programme for eligible women Incentives to community health workers have to be included in the budget for collection of samples during their home visits
Transportation of the samples	The knowledge about the terrain, cost factors and the structure of health system is the essence of the successful implementation of population-based screening programme
Linkages to treatment	Capacity building of the healthcare providers in secondary and tertiary level facilities for confirmation of diagnosis and treatment of precancers and cancer of cervix is mandatory to complete the continuum of care Screen and treat strategy minimises the loss to follow-up of screen positive women
